# Knowledge Mapping of Immunotherapy for Hepatocellular Carcinoma: A Bibliometric Study

**DOI:** 10.3389/fimmu.2022.815575

**Published:** 2022-01-31

**Authors:** Jianming Shen, Hui Shen, Lixin Ke, Jialin Chen, Xi Dang, Baoxian Liu, Yunpeng Hua

**Affiliations:** ^1^ Hepatobiliary and Pancreatic Center, the First Affiliated Hospital, Sun Yat-sen University, Guangzhou, China; ^2^ Department of Medical Ultrasonics, the First Affiliated Hospital, Sun Yat-sen University, Guangzhou, China

**Keywords:** HCC, Immunotherapy, Bibliometric study, VOSviewer, Citespace

## Abstract

**Background:**

Hepatocellular carcinoma (HCC) is one of the most common malignant tumors, and many patients are diagnosed with advanced disease. The treatment of advanced liver cancer has made significant strides in recent years, owing to the practice of immunotherapy drugs. Numerous studies have been published on immunotherapy for HCC; however, no relevant bibliometric study has been published. This study aims to gain a better understanding of the current situation and to identify potential new research directions by conducting a bibliometric analysis on immunotherapy for HCC.

**Methods:**

We searched the Web of Science Core Collection (WoSCC) for articles related to immunotherapy for HCC. Three software (VOSviewer, CiteSpace, and python) were primarily used to assess the contribution and co-occurrence relationships of various countries/regions, institutes, journals, and, authors as well as to identify research hotspots and promising future trends in this research field.

**Results:**

A total of 1,641 English articles published between 2011 and 2020 were collected, with the number of articles increasing nearly every year. The majority of publications originated from China (*n* = 893, 54.42%), followed by the United States and Japan. The Sun Yat-sen University contributed the most publications (*n* = 97, 5.91%). Nakatsura Tetsuya (*n* = 26) and Llovet JM (*n* = 366) were ranked first in the top ten authors and co-cited authors. *Cancer Immunology Immunotherapy* was the most productive academic journal on immunotherapy for HCC [*n* = 46, 2.80%; impact factor (IF) 2020 = 6.9679]. Aggregation and identification of critical nodes in the co-cited network demonstrated a shift in the field of HCC immunotherapy. Initially, the hotspots were predominantly “glypican-3”, “cytokine-induced killer cells”, and “ny-eso-1”, while the emphasis has shifted in recent years to “landscape”, “camrelizumab”, “combination therapy”, and “immune score”.

**Conclusion:**

Increased attention has been paid to HCC with the advancement of immunotherapy. At the moment, the most active frontiers are focused on better understanding the immunological landscape of liver cancer, screening the population that can benefit from immunotherapy, and the clinical application of immune checkpoint inhibitors, particularly in combination with other therapeutic options (such as local therapy and targeted therapy).

## Introduction

Hepatocellular carcinoma (HCC) is the sixth most commonly diagnosed cancer and the fourth leading cause of cancer-related deaths, worldwide (1). Additionally, the majority of HCC patients are already at an intermediate-advanced stage when their diagnosis is confirmed, necessitating palliative care (2).

Tumor immunotherapy, as a novel and successful therapeutic strategy, has a broad prospect in advanced HCC. While there are diverse immunotherapeutic approaches available for treating HCC, including immune checkpoint inhibitors, peptide vaccines, dendritic cell vaccines, chimeric antigen receptor T cells, and oncolytic viruses (3), immunotherapy trials utilizing immune checkpoint inhibitors have emerged as a major focus of research for cancer treatment (4). Immune checkpoint inhibitors (ICIs) are monoclonal antibodies directed primarily against several immune checkpoint proteins, including cytotoxic T-lymphocyte antigen 4 (CTLA-4), programmed cell death protein 1 (PD-1), and its ligand (PD-L1) in the tumor microenvironment (TME). In terms of liver cancer treatment, PD-1 antibodies significantly improved the prognosis of patients with advanced liver cancer, achieving an objective response rate (ORR) of 17%–20% and a complete remission in some patients (5, 6). Given that ICI monotherapy appears to be effective only in a small subset of patients, the search for predictors of response to ICIs and combination therapy in patients with unresectable HCC has steadily gained interest (7). Additionally, targeted drugs in combination with ICIs have a much higher ORR in advanced liver cancer, indicating a favorable therapeutic potential, and the FDA has also approved it as a first-line treatment for unresectable or metastatic liver cancer (8–10). Therefore, immunotherapy may represent a significant novel concept and a promising future perspective in the study of HCC and become an indispensable part of the treatment regimen for HCC in the near future (11).

Over the last few decades, an increasing number of studies have been published on immunotherapy for HCC. There is, however, no literature to assess the published related literature systematically. Bibliometrics can use mathematical and statistical methods to quantitatively analyze a large number of documents in a particular research field, revealing numerous facets and research trends in that field (12, 13). At present, scientometric analysis of the literature is primarily performed using CiteSpace (14), VOSviewer (15), and HistCite (16). Numerous researchers have used this strategy to assess their respective research domains (17–20).

Nevertheless, no specific scientometric research on the knowledge mapping of HCC immunotherapy has been conducted to date. The present study evaluates the literature on immunotherapy for HCC from 2011 to 2020 to describe the current state of the field and identify new research directions.

## Materials and Methods

### Data Collection

On September 27, 2021, we searched for relevant literature between 2011 and 2020 in the field of immunotherapy for HCC using the Web of Science Core Collection (WoSCC). The Science Citation Index-Expanded (SCI-E) was used as the data source with the publication types were limited to “article”. The main search terms were “primary liver carcinoma”, “primary liver cancer”, “immunotherapy”, and “immunotherapeutic”. The detailed search strategy is presented in the **Supplementary Material**. Two authors (HS and JS) independently searched the WoSCC database for relevant literature and downloaded the relevant information (title, keyword, author information, abstract, reference, *etc.*) in TXT format. Subsequently, the two authors (LK and JC) excluded articles that did not adhere to the aforementioned criteria. Divergent viewpoints would be resolved through discussions or a third party (BL and YH).

### Statistical Methods

Microsoft Office Excel 2019 (Microsoft, Redmond, Washington, USA) was used to process the data and construct a polynomial regression model (f(x)=p0​x^n^+p1​x^n−1^+p2​x^n−2^+p3​x^n−3^+…+pn​) to predict the number of articles published in 2021. Python (Python Software Foundation, Wilmington, DE) was used to draw visual maps that intuitively depict the national distribution of publications. VOSviewer (1.6.11) was used to explore collaboration networks between authors/institutes/countries/journals. In VOSviewer, nodes were used to represent countries, institutions, journals, and authors, and their size was determined by their co-occurrence frequency in titles and abstracts (21). CiteSpace 5.7.R5 (Chaomei Chen, Drexel University, USA) can extract keywords and references from publications with high citation bursts and construct a dual-map overlay for journals. Therefore, CiteSpace can be used to investigate the research trends in a certain topic (22). CiteSpace parameters included were as follows: link retaining factor (LRF = 3), e for top N (e = 2), time span (2011–2020), years per slice (1), look back years (LBY = 8), links (strength: cosine, scope: within slices), selection criteria (g-index: k = 25), and minimum duration (MD = 1).

## Results

### Annual Growth Trend of Publications

**Figure 1:**
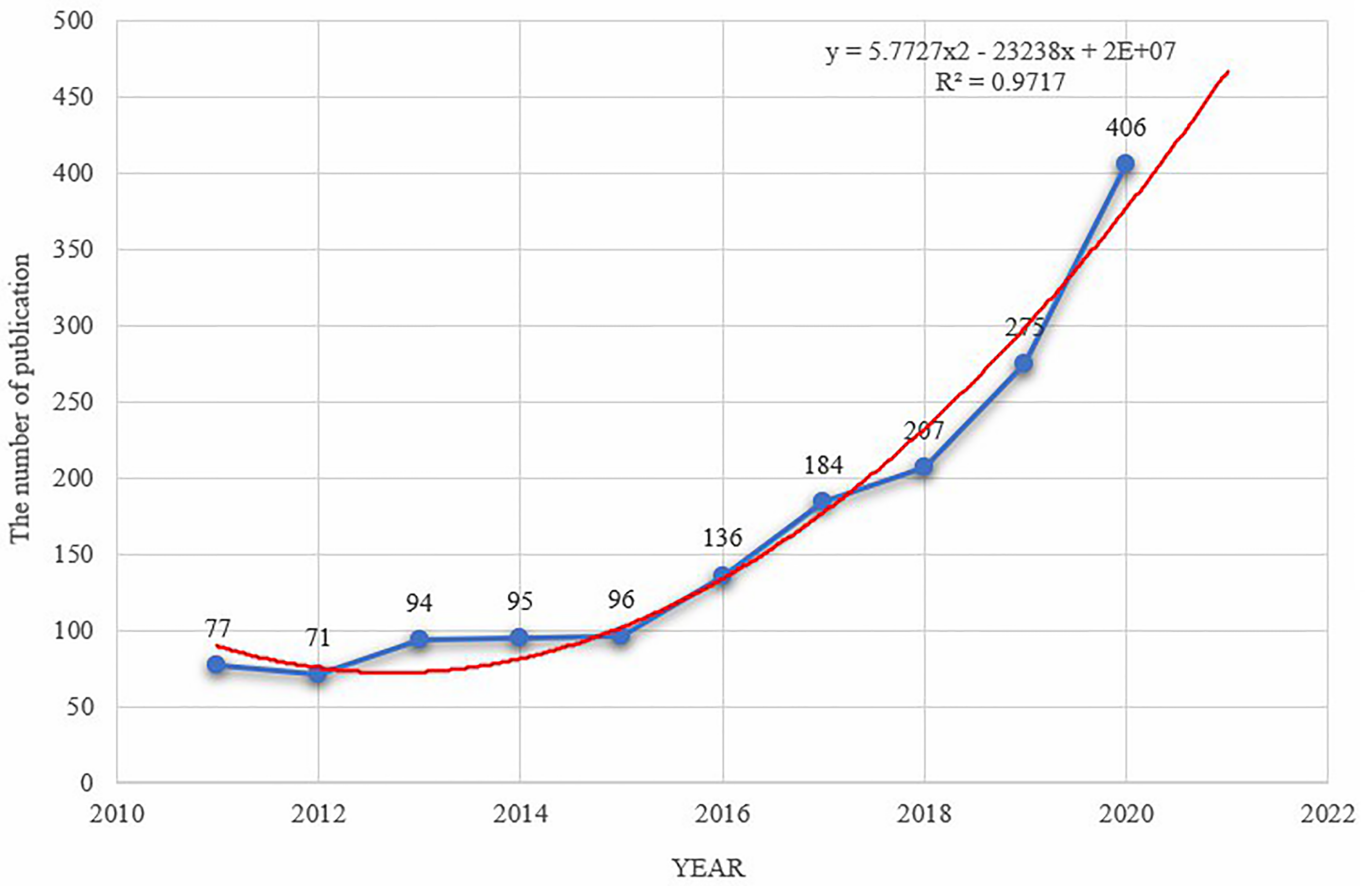
The polynomial curve fitting of publication growth in immunotherapy of HCC.

A total of 1,641 articles about immunotherapy for HCC that were published between 2011 and 2020 were obtained after searching the WoSCC database. As shown in **Figure 1**, the annual output has been increasing steadily since 2015, reaching a peak in 2020 (*n* = 406, 24.74%). The year 2012 had the lowest number of articles published (*n* = 71, 4.33%), and the annual average number of articles published was 164. By fitting the data, we observed a statistically significant relationship between the year and the number of publications (*R* ^2^ = 0.9717). According to the fitting curve, we estimated that the number of publications about immunotherapy for HCC would reach 470 in 2021.

### Countries/Regions and Institutions Analysis

**Figure 2:**
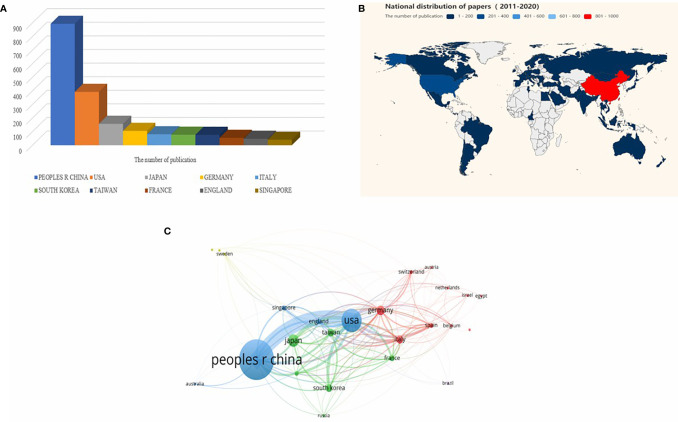
Analysis of countries/regions engaged in immunotherapy research in relation to HCC. **(A)** The top 10 most productive countries/regions. **(B)** The distribution of countries in terms of publications. **(C)** A network map showing countries/regions involved in the research on immunotherapy in relation to HCC.

A total of 1,641 articles were from 58 countries. According to the publishing number, as shown in the bar graph (**Figure 2A**) and the country distribution map (**Figure 2B**), the top 3 countries/regions were China (*n* = 893, 54.42%), United States (*n* = 392, 23.89%), and Japan (*n* = 157, 9.57%), respectively. Moreover, extensive cooperation between many countries/regions was also observed in **Figure 2C**, most notably between China and the United States.

**Figure 3:**
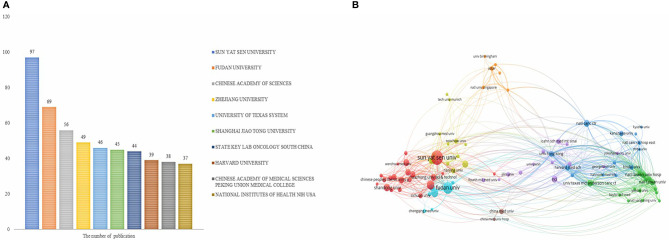
Analysis of institutions involved in immunotherapy research in relation to HCC. **(A)** The top 10 institutions involved in immunotherapy research in relation to HCC. **(B)** A network map showing institutions involved in immunotherapy research in relation to HCC.

These articles were contributed by 1,815 institutions, with the top 10 institutions contributing a total of 520 articles, accounting for 28.65% of all the articles (**Figure 3A**). Additionally, China was represented by seven of the top 10 institutions. Sun Yat-sen University ranked first (*n* = 97, 5.91%), followed by Fudan University (*n* = 69, 4.21%). As shown in **Figure 3B**, a collaboration between agencies was more extensive than that between countries. Sun Yat-sen University collaborated closely with many Chinese universities and research centers, and also with institutions from Singapore, the United States, and other countries.

### Authors and Co-Cited Authors

**Figure 4:**
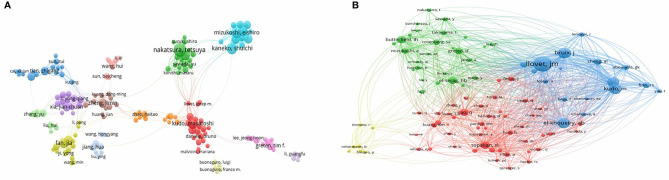
A network map showing authors **(A)** or co-cited authors **(B)** involved in immunotherapy research in relation to HCC.

More than 10,000 researchers were involved in HCC immunotherapy-related research. Among these, the top three authors with the most publications were Nakatsura Tetsuya (*n* = 26), Kaneko Shuichi (*n* = 20), and Fan Jia (*n* = 19) (**Table 1**), respectively. Among the top 10 co-citation authors (**Table 1**), Llovet JM (*n* = 366) ranked first, followed by El-Khoueiry AB (*n* = 269), and Bruix J (*n* = 260). VOSviewer was used to investigate co-authorship and citation networks between authors (**Figure 4**). Each node on the graph represents each author, the size of the circle reflects the number of articles published by the researcher, and the lines connecting the circles represent co-occurrence relationships between the authors. There was a close co-occurrence relationship between authors and co-cited authors, with more prolific authors often co-occurring more with other authors (**Figure 4**).

**Table 1:** The top 10 authors and co-cited authors involved in research on immunotherapy in relation to HCC.

Rank	Author	Count	Co-cited Author	Count
1	Nakatsura Tetsuya	26	Llovet JM	366
2	Kaneko Shuichi	20	EL-Khoueiry AB	269
3	Fan Jia	19	Bruix J	260
4	Mizukoshi Eishiro	19	Zhu AX	181
5	Kudo Masatoshi	18	Kudo M	171
6	Yoshikawa Toshiaki	18	Gao Q	158
7	Zhou Jian	17	El-Serag HB	158
8	Zheng Limin	16	Topalian SL	158
9	Arai Kuniaki	15	Cheng AL	128
10	Yamashita Tatsuya	15	Greten TF	127

### Journals and Co-Cited Academic Journals

**Figure 5:**
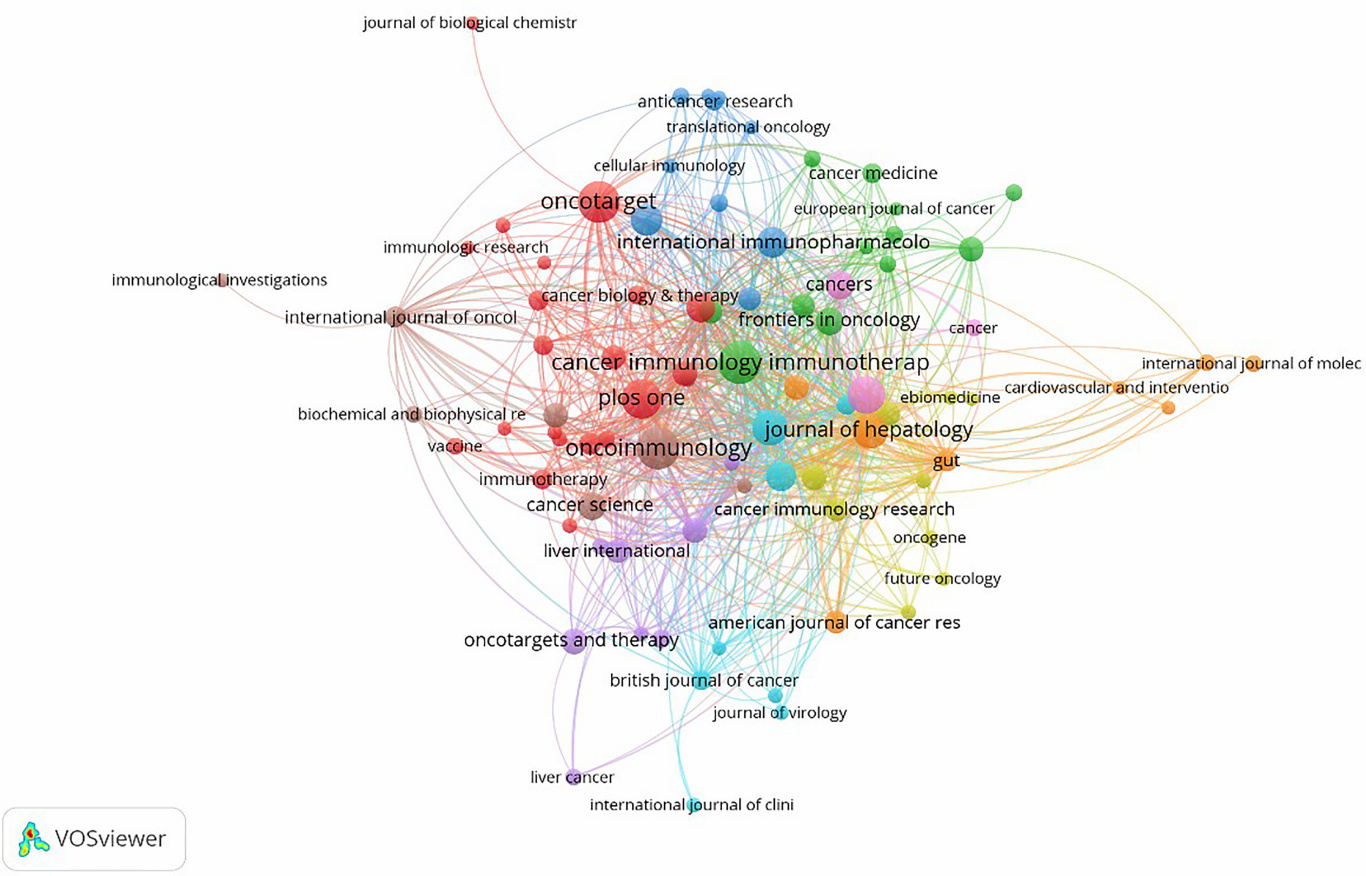
A network map showing academic journals publishing research on immunotherapy in relation to HCC.

There were a total of 466 academic journals publishing immunotherapy-related articles for liver cancer, with *Cancer Immunology Immunotherapy* (*n* = 46, IF 2020 = 6.9679) ranking first, followed by *Oncoimmunology* (*n* = 46, IF 2020 = 8.1097). Among the top ten journals (**Table 2**), 40% (4/10) were from the United States, followed by 20% (2/10) from the Netherlands and the United Kingdom. Simultaneously, among journals with more than 30 articles, the *Journal of Hepatology* (*n* = 31, IF 2020 = 25.0843) had the highest impact factor, followed by *Hepatology* (*n* = 31, IF 2020 = 17.4243). As shown in **Figure 5**, there were positive citation relationships between different journals.

**Table 2:** The top 10 academic journals involved in immunotherapy research in relation to HCC.

Rank	Journal	Count	Percent	Country	IF (2020)#
1	*Cancer Immunology Immunotherapy*	46	2.80%	Germany	6.968
2	*Oncoimmunology*	46	2.80%	United States	8.110
3	*Oncotarget*	42	2.56%	United States	NA
4	*PLOS ONE*	39	2.34%	United States	3.240
5	*Journal for Immunotherapy of Cancer*	35	2.13%	United Kingdom	13.752
6	*Hepatology*	31	1.89%	United States	17.424
7	*Journal of Hepatology*	31	1.89%	Netherlands	25.084
8	*Oncology Letters*	25	1.52%	Greece	2.968
9	*International Immunopharmacology*	24	1.46%	Netherlands	4.932
10	*Scientific Reports*	22	1.34%	United Kingdom	4.379

#IF, impact factor.

**Figure 6:**
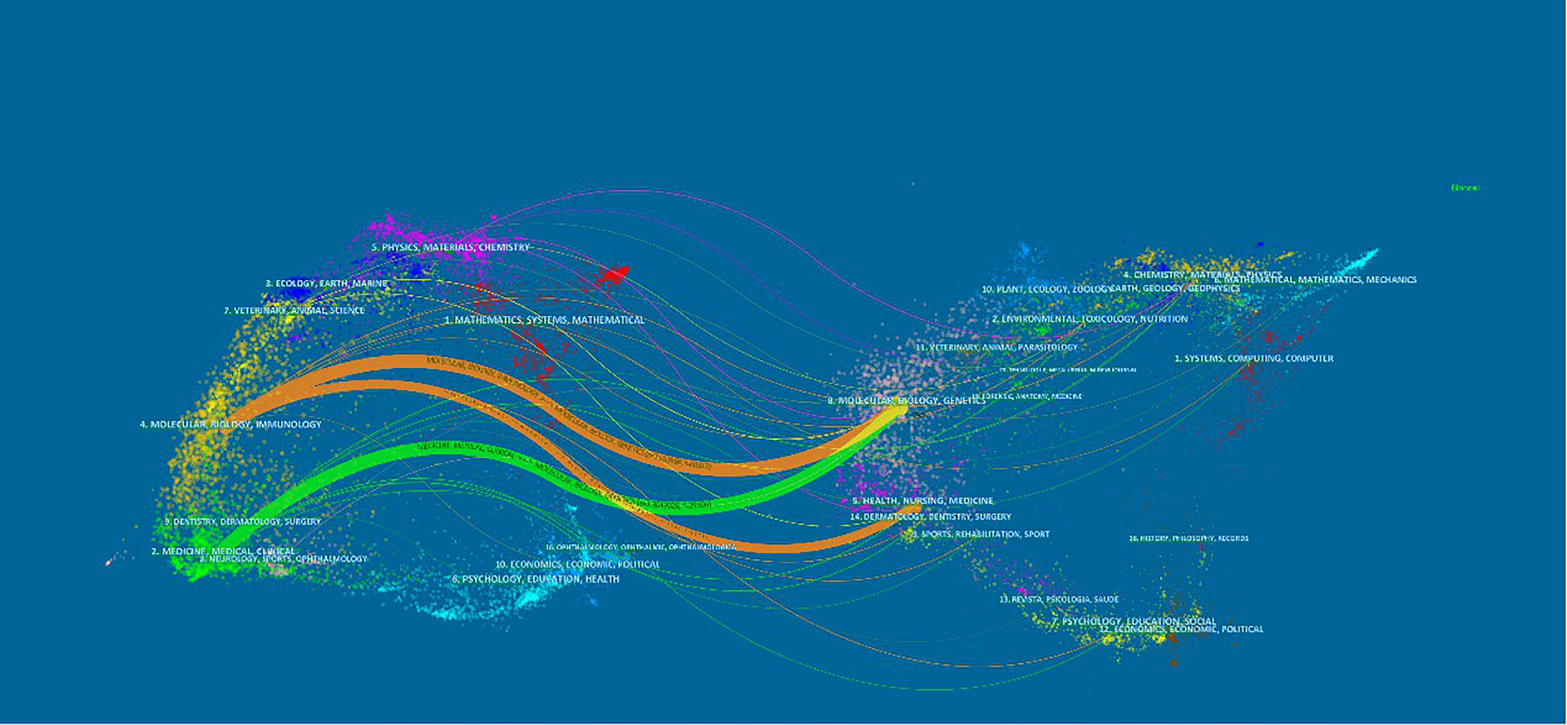
A dual-map overlay of journals related to research on immunotherapy in HCC.

The dual-map overlay of journals in **Figure 6** demonstrated the topic distribution of the journals. The citing journals were located on the left of the map, while the cited journals were located on the right. The labels represented the disciplines covered by the journals. From left to right, the colored lines depicted the citation paths. There were three distinct citation paths. Two orange citation paths suggested that studies from Molecular/Biology/Genetics journals and Health/Nursing/Medicine journals were frequently cited in studies from the Molecular/Biological/Immunological journals. A green path suggested that studies from the Molecular/Biological/Genetic journals were frequently cited from studies in Medical/Medicine/Clinical journals.

### Analysis of Co-Cited References

**Table 3** summarizes the top ten co-cited references. Among them, the paper entitled “Nivolumab in patients with advanced hepatocellular carcinoma (CheckMate 040): an open-label, non-comparative, phase 1 non-comparative 2 dose-escalation and expansion trial” published by El-Khoueiry AB et al. had the most co-citations (*n* = 256). Additionally, the literature with a total co-citation number of more than 100 (*n* = 4) was entirely composed of articles published in *Lancet* or one of its sub-journals.

**Table 3:** The top 10 co-cited references involved in research on immunotherapy in HCC.

Rank	Co-cited reference	Count	Type	IF (2020)#
R-1	El-Khoueiry AB, 2017, *Lancet*, V389, P2492	256	Clinical Trial	79.321
R-2	Bruix J, 2017, *Lancet*, V389, P56	117	Clinical Trial	79.312
R-3	Zhu AX, 2018, *Lancet Oncol*, V19, P940	108	Clinical Trial	41.316
R-4	Kudo M, 2018, *Lancet*, V391, P1163	102	Clinical Trial	79.312
R-5	Topalian SL, 2012, *New Engl J Med*, V366, P2443	94	Clinical Trial	91.245
R-6	Bray F, 2018, *CA-Cancer J Clin*, V68, P394	88	Epidemiological Study	508.702
R-7	Sangro B, 2013, *J Hepatol*, V59, P81	86	Clinical Trial	25.083
R-8	Pardoll DM, 2012, *Nat Rev Cancer*, V12, P252	75	Review	60.716
R-9	Prieto J, 2015, *Nat Rev Gastro Hepat*, V12, P681	74	Review	46.802
R-10	Ferlay J, 2015, *Int J Cancer*, V136, P0	73	Epidemiological Study	7.396

#IF, impact factor.

**Figure 7:**
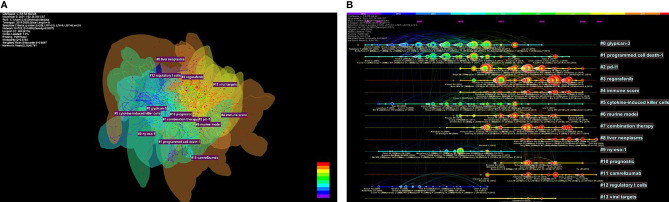
The knowledge map **(A)** and the timeline view **(B)** of references related to research on immunotherapy in HCC.

**Figure 8:**
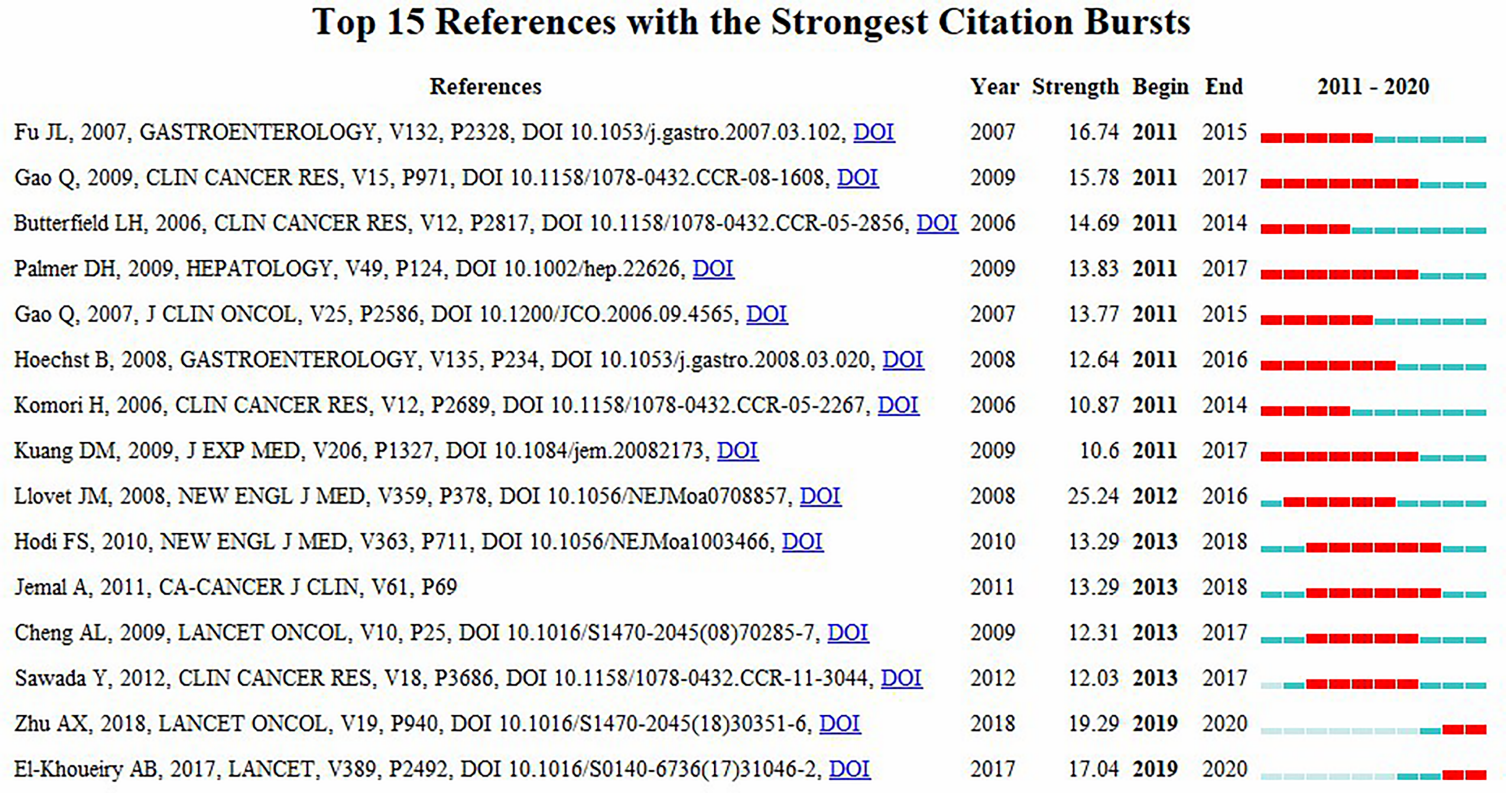
Top 15 references with strongest citation bursts.

CiteSpace was used to construct the network of co-cited references, and cluster analysis revealed 14 clusters (**Figure 7A**). Modularity Q (0.6764) and Mean Silhouette (0.761) values were both greater than 0.5. The first cluster label on the knowledge map was “#0 glypican-3” and the second cluster label was “#1 programmed cell death-1”. Simultaneously, we constructed a timeline display of co-cited references (**Figure 7B**). The timeline view is a methodology for visualizing data that combine clustering and time slicing techniques. Cluster labels are sorted according to whether they appear early or late after clustered, which not only illustrates the distribution of topics in this field but also displays the trend and interrelationship of study topics over time. In the Timeline view, different colors of nodes on the same line indicate different years. Therefore, the nodes on the left represent older references, while the nodes on the right represent more recent references. A straight line in the same horizontal position indicates the set of all clustered references belonging, and the cluster label is located at the line’s rightmost end. The closest clusters on the timeline were “#2 pd-l1”, “#3 regorafenib”, “#4 immune score”, “#6 murine model”, “#7 combination therapy”, “#8 liver neoplasms”, “#10 prognostic”, and “#11 camrelizumab”. CiteSpace was used to assess the references with a high citation burst. Citation bursts indicated that a reference had been widely cited over time and that the study findings of the references were well known in this field (**Figure 8**). We found that among the top 15 references with the strongest citation bursts, “Zhu AX, 2018, LANCET ONCOL, V19, P940, DOI 10.1016/S1470-2045(18)30351-6” (2019-2020, strength 19.29) and “El-Khoueiry AB, 2017, LANCET, V389, P2492, DOI 10.1016/S0140-6736(17)31046-2” (2019-2020, strength 17.04) were the recent emergence of high-citation references.

### Analysis of Keywords

**Figure 9:**
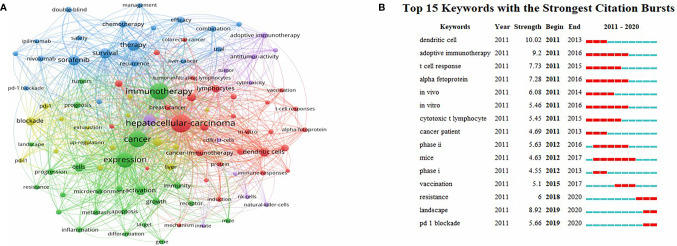
**(A)** A network map of keywords. **(B)** The keywords with the strong citation bursts in articles related to immunotherapy research in HCC.

The VOSviewer was employed to construct a network map of keywords (**Figure 9A**), and keywords with strong citation explosion were determined through CiteSpace (**Figure 9B**). In **Figure 9B**, the green line represents the time period from 2011 to 2020, while the periods of each burst keyword are plotted by the red line. The keywords that had citation bursts after 2015 were “vaccination’’ (2015–2017, strength 5.1), ‘‘resistance’’ (2018–2020, strength 6), ‘‘landscape’’ (2019–2020, strength 8.92), and ‘‘pd 1 blockade’’ (2019–2020, strength 5.66).

## Discussion

### General Information

In the present study, we performed a systematic literature search of the Web of Science databases for articles published in the last decade about immunotherapy for HCC (2011–2020). After excluding studies that did not meet the screening criteria, this scientometric study comprised 1,641 English papers published in 466 journals with 77,338 co-cited references from 1,815 institutions in 58 countries/regions.

As can be observed from the result, there is a general upward trend in the number of HCC immunotherapy-related publications, indicating that this topic has received significant attention in recent years. Additionally, three key inflection points occurred in the years 2015, 2018, and 2019. This phenomenon may be connected to significant events in the field of HCC immunotherapy. The US Food and Drug Administration (FDA) approved PD-1 checkpoint inhibitors (nivolumab and pembrolizumab) for use in non-small cell lung cancer and melanoma at the end of 2014, ushering in a new era of immunotherapy research for HCC. On September 30, 2017, the FDA authorized nivolumab for treatment in patients with advanced HCC (5). A year later, cancer immunotherapy using checkpoint inhibitors was awarded the Nobel prize. Following this, research on HCC immunotherapy-related research has advanced to a new level, and its clinical application has been a research hotspot in recent years.

While China was the only developing country to rank in the top 10 most productive countries/regions, it accounted for more than half of all publications. The explanation for this could be that China accounted for 55% of all liver cancer cases globally, indicating that China is a country with a high incidence of liver cancer (23, 24). Institutional distribution was generally consistent with the country distribution. China contributed seven of the top 10 institutions, followed by the USA with three institutions. Close collaborations were observed between countries/regions and institutions; however, collaborations between agencies were found to be stronger than those between countries, implying that international collaborations should be strengthened (**Figure 3B**).

At least 15 articles were published by each of the top ten active authors. All of the top 10 active authors were from Asia, including 7 Japanese and 3 Chinese scholars. The finding may imply that Asian researchers play a significant role in HCC immunotherapy research and have made significant contributions. When co-cited authors are taken into account, the top 10 authors with at least 127 co-citations made significant contributions to the field of HCC immunotherapy. Llovet JM (366 co-citations) ranked first, followed by Anthony B El-Khoueiry (269 co-citations) and Bruix J (260 co-citations). Llovet JM is well known for his significant contributions to the formulation of liver cancer guidelines (25), the exploration of liver cancer treatment methods (26), and immunotherapy mechanisms (27). Anthony B El-Khoueiry has chaired several clinical trials evaluating immunotherapy drugs for the treatment of advanced liver cancer. CheckMate-040 (5) played a critical role in the FDA’s approval of Opdivo (nivolumab) for the treatment of primary liver cancer patients who have failed to respond to sorafenib or have developed intolerance. Bruix J focused his research on targeted drugs for the treatment of liver cancer and contributed to the development of HCC management guidelines (28, 29), which were important in influencing the direction of HCC immunotherapy research.

Only 341 publications were published in the top ten academic journals, accounting for only 20.78% of all articles. *Cancer Immunology Immunotherapy* (30) ranked first in terms of total publications, followed by *Oncoimmunology* (30) and *Oncotarget* (31), indicating that these journals were particularly interested in articles regarding HCC immunotherapy research. These data will aid future scientists in selecting journals when submitting HCC immunotherapy-related manuscripts.

Furthermore, after comprehensively analyzing the data of authors, co-cited authors, and co-cited references, we found that Masatoshi Kudo was the only scholar who appeared in all three indicators, suggesting that he is an accomplished author in this field, and his teams would make excellent potential collaborators for researchers.

### Knowledge Base

When two publications are cited jointly by a third citing publication, this is referred to as a co-citation relationship (32). The more often a piece of literature is cited, the more significant it is perceived in a certain field. Therefore, the most frequently cited publications or high-impact literature can be viewed as a knowledge base and primary focus for researchers in a particular field. As shown in **Table 3**, they are mainly from top-ranked journals and composed of six clinical trials, two reviews, and two epidemiological studies.

Firstly, these six clinical studies provide an overview of the discovery and development process for HCC immunotherapy. Sorafenib ushered in the era of targeted therapy for liver cancer in 2007. Over the last 10 years, the exploration of targeted therapy for liver cancer was mostly stagnant, although immunotherapy showed promise. Topalian SL et al. (33) (R-5) conducted a multicenter phase I clinical trial (NCT00729664) in 2012 to evaluate the efficacy of anti-PD-L1 monoclonal antibodies in a variety of advanced solid tumors. Although these patients enrolled in this study did not include liver cancer patients, the results demonstrated the safety and activity of anti–PD-L1 antibodies in patients with advanced cancer, prompting the scholars to investigate the use of anti-PD-L1 in the treatment of advanced liver cancer. One year later, Sango et al. (34) (R-7) published the results of a clinical trial using CTLA-4 antibody (tremelimumab) to treat HCC. The clinical trial showed that the CTLA-4 antibody exhibited antitumor and antiviral activity, with manageable adverse drug reactions. Over the next few years, pertinent clinical research was conducted. El-Khoueiry AB et al. (5) (R-1) published the most co-cited study (CheckMate040) in 2017, with 268 co-citations. This study reported the use of an anti-PD-1 antibody (nivolumab) to treat advanced HCC. The results showed that, regardless of whether or not they had received sorafenib treatment, nivolumab had a favorable response rate and overall survival time, and the drug was safe and tolerable. Simultaneously, another clinical trial with a novel targeted drug, regorafenib, achieved favorable findings the same year. Bruix J et al. (35) (R-2) reported this study (NCT01774344). The FDA approved regorafenib in April 2017 for patients with hepatocellular carcinoma who have previously received sorafenib treatment. Another clinical trial (Keynote-224) (NCT02702414) (R-3) published in 2018 by Zhu AX et al. (6) evaluated the safety and effectiveness of anti-PD-1 drug Pembrolizumab in 104 patients with advanced HCC who had previously received sorafenib. Similarly, Kudo M et al. (36) (R-4) presented the results of the REFLECT trial (NCT01761266), which compared overall survival in patients treated with lenvatinib against sorafenib as a first-line treatment for unresectable hepatocellular carcinoma. The FDA approved lenvatinib as a single-agent first-line treatment for patients with unresectable liver cancer based on the results of the REFLECT trial.

GLOBOCAN is a significant project of the International Agency for Research on Cancer (IARC), which tracks the cancer incidence, mortality, and cancer development trends of 36 types of cancer in 185 countries/regions worldwide, to summarize the global epidemiology of cancer and its impact on human health. In 2012 and 2018, Ferlay J et al. (37) (R-10) and Bray F et al. (24) (R-6) reported on cancer incidence and mortality worldwide, respectively. These two articles contributed significant epidemiological data on hepatocellular cancer, as evidenced by the fact that they were cited in several publications. In 2012 and 2015, Pardoll DM et al. (38) (R-8) and Prieto J et al. (39) (R-9) published two high-quality reviews on liver cancer immunotherapy, respectively. Both of them provided a complete overview of the immunotherapy mechanism for liver cancer at that time.

In general, these ten highly co-cited references showed the current epidemiology of liver cancer, the exploration, and development of immunotherapy for advanced liver cancer, as well as the clinical application of these treatments. Co-citation analysis can provide us with a wealth of useful information, allowing us to gain a better understanding of the evolution of the knowledge structure relating to HCC immunotherapy.

### Research Hotspots

To further investigate and describe new immunotherapy hotspots for HCC, we used CiteSpace to examine the co-cited reference. As shown in **Figure 7**, early research focused on the investigation of various immunotherapy methods for HCC, such as “#0 glypican-3”, “#5 cytokine-induced killer cells”, and “#9 ny-eso-1”, whereas current studies focused on “#3 regorafenib”, “#7 combination therapy”, and “#11 camrelizumab”. Simultaneously, the present stage focuses on how to screen patients who react to immunotherapy, e.g., the “#4 immune score”.

Targeted therapy is a critical component of treatment for patients with advanced liver cancer, and regorafenib is one of the representative drugs (40). Although sorafenib has been on the market for more than a decade, the overall therapeutic result is insufficient, with approximately 30% of patients benefiting and these people typically developing drug resistance within 6 months (41). Regorafenib was approved by the FDA in 2017 as the second-line standard treatment for patients with advanced HCC after it was demonstrated to be efficacious in HCC patients with sorafenib resistance (35). Subsequent clinical studies on regorafenib demonstrated that it is a good and relatively safe treatment option for patients with advanced hepatocellular carcinoma (sorafenib resistance) (42). This may explain why regorafenib has become a study priority for academics over the last 2 years. Simultaneously, researchers investigated more effective treatments. Tasuku Honjo, a Japanese researcher, discovered the T-cell suppressor receptor PD-1 in 1992, paving the way for the development of negative immunoregulatory cancer therapies (43). Camrelizumab is one of the most representative drugs. Catilizumab was approved as a second-line drug for the systematic treatment of liver cancer by the National Medical Products Administration (NMPA) in 2020 based on the findings of various clinical trials (31). This adds a new treatment option to the treatment options for patients with advanced liver cancer. Clinical trials on immune checkpoint inhibitors are currently being conducted in large numbers, and the treatment of liver cancer has also come to the immune era (44). This may possibly be why Catilizumab has developed a reputation as a research hotspot. However, immune checkpoint inhibitors are likely to have limited activity as monotherapy in the majority of unselected HCC patients, which makes researchers consider the prospect of combined therapy (45). Anti-VEGF antibodies normalize tumor blood vessels, allowing T cells to invade tumors more effectively, while VEGF inhibitors can reprogram TME into immunostimulatory environments (30, 46). Antibodies against PD-1 and PDL-1 can increase T cell’s ability to target tumors. These mechanisms provide a theoretical basis for the combination of targeted therapy and immunotherapy. The results of IMbrave150 demonstrate that Atezolizumab combined with bevacizumab is a reasonable treatment option for patients with advanced unresectable liver cancer (47). Subsequently, the FDA approved atezolizumab in combination with bevacizumab as a first-line therapeutic option for patients with advanced unresectable liver cancer. These findings aroused researchers’ enthusiasm for the exploration of combined therapies, and novel combinations of various therapies have been proposed, including PD-1/PD-L1 immunotherapy in combination with CTLA-4 immune checkpoint inhibitors, transhepatic artery chemoembolization (TACE), or hepatic artery infusion chemotherapy (HAIC) in combination with immune checkpoint inhibitors or targeted therapy, and others (48, 49). Without a doubt, combination therapy is a current research hotspot.

Unfortunately, not all patients with HCC benefit from immunotherapy or combination therapy, which may be due to differences in their immunological environments (50). The TME is composed of immune cells, stromal cells, endothelial cells, inflammatory mediators, and extracellular matrix molecules, with immune cells and stromal cells being the two major non-tumor components (51). Stromal and immune scores could be calculated by estimation of stromal and immune cells in malignant tumor tissues using expression data (ESTIMATE) (52). Immune scores are an excellent marker for predicting neoplastic outcomes and guiding clinicians in making treatment decisions (53). Therefore, it is critical to develop reasonable and accurate biomarkers for immunotherapy in combination with other therapies, to attain precision medical treatment.

Additionally, we used CiteSpace to identify burst keywords, which may serve as an essential indicator of research hotspots or research frontiers over time. As shown in **Figure 9B**, the evolution of burst keywords over the past decade demonstrates the field’s continued progress in HCC immunotherapy research. Based on this, we found that the keyword “landscape” has been confirmed as the strongest burst keyword in 2019. The term “landscape” refers primarily to the immunological and metabolic landscapes associated with cancer. As is well known, cancer immunotherapy is a relatively new and promising treatment modality that has emerged in recent years. While this treatment is capable of producing significant clinical effects, the tumor types were diverse and heterogeneity was unavoidable. Therefore, establishing a systematic and detailed tumor immunological landscape can aid in the development of tumor immunotherapy, the identification of effective targets and biomarkers (54), and the understanding of immune escape and drug resistance (55).

Bibliometric study is a method that depicts the evolution of scientific knowledge and its structural relationships, illustrating numerous implicit complicated relationships among clusters of knowledge (56). Thus, by comprehending these intricate knowledge relationships, researchers can understand the trend of knowledge in a specific field. Our bibliometric study elucidated that the discovery of additional immune targets, the elucidation of immune characteristics of the immune microenvironment in liver cancer, the exploration of biomarkers for predicting immunotherapy effect, and increased attempts to combine treatments may play a significant role in liver cancer immunotherapy in the following years.

## Strengths and Limitations

For the first time, our study conducted a systematic analysis of HCC immunotherapy publications and their trend in an intuitive, objective, and accurate manner, which could serve as a comprehensive guide for clinicians and scholars working in this field. Simultaneously, we used a variety of bibliometrics software, to investigate research hotspots in multiple dimensions. Inevitably, there are some limitations in this study. First, the literature included in our study may not be exhaustive. On the one hand, our study investigated data from the WoSCC exclusively; data from other significant search engines such as PubMed, Embase, and Ovid were excluded. On the other hand, because the articles retrieved were limited to those published in English, some linguistic bias was introduced. Thus, the articles identified may not adequately reflect represent all HCC immunotherapy research. Second, due to their low citation rate, recently published high-quality articles may not receive the attention they deserve. This also demonstrates the importance of future research updates. Finally, while publications in the year 2021 were excluded due to insufficient data, this study includes the vast majority of papers published in the field of HCC immunotherapy between 2011 and 2020; new data may have few effects on the final results.

## Conclusion

In general, advanced liver cancer treatment has entered a personalized precision treatment phase. Understanding the immune context of liver cancer by detecting the immune landscape of the disease, screening the population who may benefit from immunotherapy by calculating immune scores, and evaluating the efficacy of combined treatment strategies, particularly combinations of immune checkpoint inhibitors and other drugs, are urgent questions that need to be addressed urgently and also the future directions of immunotherapy research for liver cancer.

## Data Availability Statement

The original contributions presented in the study are included in the article/**Supplementary Material**. Further inquiries can be directed to the corresponding authors.

## Author Contributions

YH, BL, JS, and HS designed this study. JS and HS performed the search and collected data. LK, XD, and JC re-checked data. JS and LK performed analysis. All authors contributed to the article and approved the submitted version.

## Conflict of Interest

The authors declare that the research was conducted in the absence of any commercial or financial relationships that could be construed as a potential conflict of interest.

The reviewer GW declared a shared parent affiliation with the author(s) to the handling editor at the time of the review.

## Publisher’s Note

All claims expressed in this article are solely those of the authors and do not necessarily represent those of their affiliated organizations, or those of the publisher, the editors and the reviewers. Any product that may be evaluated in this article, or claim that may be made by its manufacturer, is not guaranteed or endorsed by the publisher.
