# Supplementary material for: Knowledge Mapping of Immunotherapy for Hepatocellular Carcinoma: A Bibliometric Study
Source: Front Immunol. 2022 Jan 31;13:815575. doi: 10.3389/fimmu.2022.815575 (PMC8841606; doi:10.3389/fimmu.2022.815575)
Supplement: Supplementary file 1 [file DataSheet_1.docx]

“primary liver carcinoma” OR “primary liver carcinomas” OR “primary hepatic carcinoma” OR “primary hepatic carcinomas” OR “primary liver cancer” OR “primary liver cancers” OR “primary hepatic cancer” OR “primary hepatic cancers” OR “hepatocellular carcinoma” OR “hepatocellular carcinomas” OR “hcc” OR “hepatic cell carcinoma” OR “hepatic cell carcinomas” OR “liver cell carcinoma” OR “liver cell carcinomas” OR “hepatic cell cancer” OR “hepatic cell cancers” OR “liver cell cancer” OR “liver cell cancers” OR “hepatocarcinoma” OR “hepatocarcinomas” OR “hepatoma” OR “liver neoplasms” OR “liver neoplasm”

“immunotherapy” OR “immunotherapies” OR “immunotherapeutic” OR “immunotherapeutics” OR “ICI” OR “ICIs” OR "CPI" OR “immune-checkpoint inhibitor” OR “immune checkpoint inhibitor” OR “immune-checkpoint blockade” OR “immune checkpoint blockade” OR “Nivolumab” OR “pembrolizumab” OR “atezolizumab” OR “avelumab” OR “durvalumab” OR “ipilimumab” OR “PD-1” OR “cytotoxic T lymphocyte-associated antigen-4” OR “CTLA-4”
